# Molecular techniques drive cutting edge advancements in management of cutaneous T cell lymphoma

**DOI:** 10.3389/fimmu.2023.1228563

**Published:** 2023-08-15

**Authors:** Mitchell N. Lefebvre, Nicholas Borcherding, Ryan J. Reis, Eric Mou, Vincent Liu, Ali Jabbari

**Affiliations:** ^1^ University of Iowa Carver College of Medicine, Iowa City, IA, United States; ^2^ Department of Dermatology, University of Iowa, University of Iowa Hospitals and Clinics, Iowa City, IA, United States; ^3^ Department of Pathology and Immunology, Washington University School of Medicine, St. Louis, MO, United States; ^4^ Cancer Biology Graduate Program, University of Iowa Carver College of Medicine, Iowa City, IA, United States; ^5^ Department of Hematology and Oncology, University of Iowa Hospitals and Clinics, Iowa City, IA, United States; ^6^ Department of Pathology, University of Iowa Hospitals and Clinics, Iowa City, IA, United States; ^7^ Iowa City Veterans Affairs Medical Center, Iowa City, IA, United States

**Keywords:** cutaneous T cell lymphoma, flow cytometry, RNA sequencing, high throughput sequencing, TCR - T cell receptor

## Abstract

Cutaneous 5T cell lymphoma (CTCL), characterized by malignant T cells infiltrating the skin with potential for dissemination, remains a challenging disease to diagnose and treat due to disease heterogeneity, treatment resistance, and lack of effective and standardized diagnostic and prognostic clinical tools. Currently, diagnosis of CTCL practically relies on clinical presentation, histopathology, and immunohistochemistry. These methods are collectively fraught with limitations in sensitivity and specificity. Fortunately, recent advances in flow cytometry, polymerase chain reaction, high throughput sequencing, and other molecular techniques have shown promise in improving diagnosis and treatment of CTCL. Examples of these advances include T cell receptor clonotyping via sequencing to detect CTCL earlier in the disease course and single-cell RNA sequencing to identify gene expression patterns that commonly drive CTCL pathogenesis. Experience with these techniques has afforded novel insights which may translate into enhanced diagnostic and therapeutic approaches for CTCL.

## Introduction

Cutaneous lymphomas are a heterogeneous assortment of clonal T-, B-, and natural killer (NK) cell malignancies that localize to the skin but may additionally involve or disseminate to the blood, lymph nodes, and viscera. Cutaneous T cell lymphomas (CTCL) comprise most cases, with neoplastic cells commonly exhibiting a CD4^+^CD8^-^ immunophenotype. The most common type of CTCL is mycosis fungoides (MF), characterized by malignant cells localizing to the dermal-epidermal junction and the epidermis as single cells and as collections (1, 2). MF, typified by pruritic patches, plaques, and tumors classically distributed over the trunk, proximal extremities, and waist and buttock region, often behaves indolently. However, a subset of patients with MF will experience a more rapidly progressive, treatment-resistant disease course (1, 3). The leukemic form of MF, Sézary syndrome (SS), most often presents with erythroderma with pruritus and lymphadenopathy (4). Because SS imparts significant morbidity and mortality (3), early, accurate diagnosis and an appreciation of factors indicative of disease progression should optimize management.

CTCL tumor cell populations typically exhibit a dominant T cell clone that shares common rearranged TCRγδ or TCRαβ chains, although CTCL cases with multiple clones have been reported (5, 6). Malignant cells also frequently exhibit genomic alterations, including the more common gene copy number alterations and less common somatic mutations (7). These mutations can result in aberrant protein expression and function such as increased JAK-STAT signaling that promotes tumor cell proliferation (8). The expansion of malignant CD4 T cells results in increased CD4:CD8 T cell ratios and the presence of atypical tumor cells in the blood (9).

CTCL management is frequently complicated by delayed diagnosis, in part because MF mimics inflammatory dermatoses such as atopic dermatitis and psoriasis at a clinical and histopathological level (9, 10). Diagnosing CTCL with traditional diagnostic tools, including clinical presentation, immunohistochemistry, and histopathology, is imperfect, resulting in a median delay in diagnosis of forty eight months in patients who presented with only cutaneous disease (11).

The accuracy and efficacy of clinical tools also remain limited when assessing individualized risk for disease progression and determining optimal targeted therapies. Diagnostic delays and prognostic errors may result in inappropriate treatment of CTCL and disease progression. Therefore, there is a clear need for new tools that the clinician can deploy in the care of CTCL patients. Scientists and clinicians previously validated a set of clinical variables to propose prediction models for disease progression (12), termed the Cutaneous Lymphoma International Prognostic Index (13). These risk factors for progression in patients are based on the physical exam, including total body surface area involvement and skin lesion morphology, e.g., the presence of skin plaques versus patches. Reliance on clinical staging is, however, of limited use in the initial stages of disease (14).

Current therapies for advanced CTCL include chemotherapy, radiotherapy, and immunomodulatory medications; however, allogeneic hematopoietic stem cell transplant in the setting of low disease burden is the only potentially curative treatment currently available (15, 16). Chemotherapy and non-chemotherapeutic therapies are frequently unable to provide durable disease control in MF/SS (17). Therefore, there is a need for novel therapies that will provide symptom relief, disease control, and disease cure. Identifying novel therapeutic targets can be challenging, in part because prominent levels of tumor heterogeneity exist within and between patients (18–20). Recent and emerging studies dissecting mutated pathways in CTCL continue to identify novel potential therapeutic targets. These include targets related to signaling pathways, cell surface proteins, and host immune responses (21).

Assessment of parameters such as tumor T cell receptor (TCR) clone frequency and CTCL somatic mutation burden, the latter of which is primarily driven by copy number alteration, offer the ability to more accurately diagnose CTCL, determine the optimal treatment of the disease, and monitor treatment response. Molecular techniques can assess these parameters. Techniques include flow cytometry, polymerase chain reaction (PCR), high throughput sequencing (HTS), and single-cell RNA sequencing (scRNA-seq) performed on patient tissue samples. In this review, we discuss how clinicians can leverage molecular techniques to aid in CTCL diagnosis, prognosis, and monitoring of treatment response. We also highlight how researchers utilize molecular techniques to increase understanding of CTCL pathogenesis and potentially identify future therapeutic targets.

## Diagnostic and prognostic evaluation of CTCL

### Diagnosis

Molecular techniques, including flow cytometry, PCR, quantitative PCR, HTS, scRNA-seq, and proteomic analysis, have shown promise in identifying CTCL tumor cells from the skin and/or blood to facilitate quicker and more dependable CTCL diagnosis as compared to clinical tools. These techniques distinguish CTCL from benign inflammatory dermatoses by exploiting tumor characteristics, including signatures of TCR clonality, gene expression, miRNA expression, and protein composition **Table 1**. It is important to consider the unique strengths and weaknesses of each approach such as sensitivity, specificity, and cost.

**Table 1:** Novel Molecular Tools with Potential for Enhancing Diagnosis, Prognosis, and Management of CTCL.

Technique	Description	Potential Role			Limitations
		Diagnosis	Prognosis	Treatment	
**Flow cytometry**	Assesses protein and transcriptional factor expression by lymphocytes	Detection of abnormal T cell protein expression	Yes, when combined with PCR and lab measurements	Can detect persistent tumor cells but loses sensitivity	Variable sensitivity especially in early blood-stage disease
**PCR**	Detects a limited number of specific DNA segments	Detection of clonal TCR	Yes, when combined with FC and labs	Can detect persistent tumor cells	Variable sensitivity and specificity
**HTS**	Reads a high number of DNA segments	Detection of clonal TCR	TCR clone frequency and genomic mutations associated with variable prognosis	Can detect exceptionally low levels of persistent tumor cells	Prohibitive cost, no standardized protocols
**scRNA-seq**	Captures the entire transcriptomic profile of individual cells	Can ID patterns within either the neoplastic or normal immune cell populations and can give TCR clonality information depending on technology used	Can classify and predict based on transcriptional info but also immune compartment wide info such as % of immune cell populations and transcriptomics of the healthy immune system	Ability to predict response to treatment based on neoplastic or normal immune cell signatures and TCR clonality	Prohibitive cost, no standardized protocols, requires specialized training to implement

Flow cytometry is often used to diagnose the presence of a malignant cell population in peripheral blood and stage the disease. Well-established guidelines inform this. Updated WHO-EORTC criteria define significant (B2) blood involvement as ≥1000/ml aberrant T cells typically with a CD4+/CD7- or CD4+/CD26- immunophenotype (22). However, benign dermatoses may also have associated abnormal CD4 T cell populations, which may limit the specificity of these markers (23, 24). Proposed alternative parameters include the presence of clonal TCRVβ chains; gating on CD4^+^CD26^-^ populations appears to increase the sensitivity of TCR-Vβ testing (25, 26).

CTCL diagnosis may be made by PCR detection of malignant TCR δ or β chains, another WHO-EORTC criteria (22, 27). Clonal PCR may facilitate the detection of early-stage MF when traditional diagnostic techniques lack sufficient sensitivity (7, 28). Strengths of PCR include higher sensitivity than flow cytometry for diagnosis and the ability to track disease from diagnosis and treatment, whereas treatment can reduce the reliability of flow cytometry and RNA based disease markers (29).

PCR is limited, however, by factors including pre-test probability, sample quality, disease stage, assay method, and primer design; as a result, it is estimated that the sensitivity of PCR among institutions varies from 50-90% (7). There is also not a clear consensus on where the cutoffs for PCR monoclonality should be set, as increasing the specificity of a PCR assay may compromise the sensitivity to an unacceptable level (30). For these reasons, it is recommended that standardized PCR assays, such as the PCR BIOMED-2 kit, be used in favor of non-standardized PCR kits to identify mutations more accurately (31). A proposed solution is to combine TCRβ and TCRγ clonality PCR assays to improve the sensitivity when detecting early MF (32). PCR specificity may be limited by the fact that autoimmune and inflammatory conditions sometimes exhibit dominant T cell clones (32). This may be addressed by determining if a shared dominant TCR clone is detected in multiple skin lesions, which is unique to CTCL (33).

HTS of malignant TCR clones holds advantages over PCR, including increased sensitivity and specificity, earlier disease and disease recurrence detection, and independence from diagnostic biases such as primer selection. HTS of the TCRγ and TCRβ genes exhibited 100% sensitivity versus 70% sensitivity for PCR analysis of paired samples in MF (34). In the same study, malignant T cells could be isolated from the blood of patients with new skin lesions but no clinical involvement of the peripheral blood, highlighting that early blood involvement may be detected by HTS. Numerous other studies have demonstrated that HTS has superior sensitivity and/or specificity as compared to PCR for detection of malignant cells in the skin and or blood (35–38).

While TCR sequencing is a powerful tool for the diagnosis of CTCL, it may sometimes be insufficient for this purpose such as is the case when CTCLs exhibit polyclonal TCR distributions. Two case reports identified patients with clinical features of CTCL but without dominant TCR clones on PCR or HTS. The researchers instead utilized somatic mutation profiling to identify tumor cell mutations in *PIKC3D* or *TP53* that were consistent with CTCL (39). Single-cell RNA sequencing determined that skin-localized CTCL cells exhibit increased expression of *TOX*, *CCR4*, and *STAT5* and decreased expression of *PSORS1C2*, among others, when compared with benign inflammatory dermatoses (40).

### Prognosis

Accurate prognostic information in CTCL is critical because it guides decisions about how to best deploy resource-intensive and potentially risky therapeutic interventions. One technique that may be complementary to clinical staging for prognosis is the assessment of multiple parameters including patient age, lactate dehydrogenase (LDH) levels, and the presence of a dominant T cell clone. Newer methods to assess progression risk include HTS to determine tumor clone frequency and identifying unique tumor cell gene expression patterns through transcriptional profiling. These techniques often provide more reliable prognostic value than clinical staging does (7).

Integrating results from flow cytometry, TCR clonotyping, and lab measurements can predict disease progression risk and overall survival in MF. Overall patient survival was predicted via a Prognostic Index Model using the four risk factors of clinical stage IV, age greater than 60 years, elevated LDH, and large-cell transformation in the skin (39). Peripheral blood TCR clonality, flow cytometry, and LDH predicted progression to advanced stage MF (41). A dominant T cell clone in peripheral blood was associated with a shorter time to systemic treatment in patients with Stage IB MF (42). Low-level blood involvement, determined by positive TCR gene rearrangement and abnormal T cell population identification on flow cytometry, was associated with decreased overall survival in patients with stage IA to IIA CTCL (43).

HTS has the potential for more accurate identification of patients with early-stage disease who are at significant risk of disease progression. Tumor clone frequency (TCF) in the skin of patients with MF, as measured by HTS of *TCRB*, is predictive of progression-free and overall survival in patients with CTCL (44). A TCF of greater than 25% was associated with a 92% positive predictive value and 83% negative predictive value for 5-year disease progression or death. The prognostic value of TCF analysis was superior to other prognostic techniques, including clinical disease staging. Higher TCF was associated with increased somatic mutation burden in CTCL cells, which may explain why increasing TCF frequency correlates with increased disease risk (44).

Transcriptional profiling of CTCL cells holds the potential for predicting disease progression and overall survival based on gene expression patterns. One study performed microarray analysis of skin tumor cells and identified three distinct gene expression clusters that predicted favorable, intermediate, and poor disease prognosis. Certain genes were preferentially expressed in favorable, such as *WIF-1*, versus poor, such as *IL-17F*, prognosis clusters (45). RNA sequencing of skin samples from patients with early-stage MF determined that downregulation of genes such as *CXCR4* and *CD69* and upregulation of genes including *HSPA1A* and *IL7R* was associated with tumor progression (46). Malignant cells from clinically unaffected skin showed similar differential gene expression, suggesting that early disease spread may be clinically undetectable. In patients with advanced, disseminated disease, as defined by SS, *IL32* overexpression portended poorer survival (2). *TOX* is overexpressed in early-stage MF, and *TOX* upregulation correlates with an increased risk of disease progression and decreased survival (47, 48). Whether these gene expression patterns correlate with advanced disease or are true drivers of progression remains unknown.

## Advancing understanding of CTCL pathogenesis

Heterogeneity in T cell clones, tumor somatic mutations, and gene expression are key features of CTCL biology. Genetic alterations in SS include copy number alterations, DNA rearrangements and fusion transcripts, single nucleotide variation, and epigenetic changes (49). Genetic alterations in MF include DNA rearrangements, copy number alterations, fusion transcripts, gene mutations, and epigenetic alterations. This heterogeneity presents a challenge to researchers and clinicians who attempt to better understand CTCL pathogenesis. Indeed, one study determined that no one dominant mutation was shared between different patients’ tumor cells (21). Recent efforts by researchers using molecular techniques such as HTS and scRNA-seq are identifying pathways that drive CTCL pathogenesis and identifying potential therapeutic targets. Understanding these is important because it offers a glimpse into the future of CTCL treatment.

A multitude of studies have deployed qRT-PCR, scRNA-seq, and HTS of isolated tumor cells to identify genes associated with CTCL disease progression and response to treatment. Numerous differentially regulated genes have been identified; however, significant heterogeneity exists in mutations found in cells from different patients with the same type of CTCL and even within different tumor cells from the same patient (50, 51). This heterogeneity complicates efforts to identify shared drivers of CTCL pathogenesis, and it may be that currently available sequencing technologies limit researchers’ abilities to identify all mutations. Mutation patterns have been identified despite these challenges. One notable signaling pathway that is aberrantly upregulated in MF and SS is JAK-STAT signaling, which appears to drive increased proliferation and activation of malignant T cells and mediate disease progression in a mouse model (52). Loss of *SOCS1* expression, which normally inhibits JAK activity, appears to drive increased JAK signaling in MF. In contrast, in SS copy number variants of the *STAT3* and *STAT5B* genes or gain of function mutations in *JAK* and *STAT* genes drive aberrant JAK-STAT signaling.

Several additional mutated genes have been linked to CTCL pathogenesis. A genomic analysis of published CTCL genomic databases identified *RLTPR* as commonly mutated in tumors, which may drive NF-κB activation and IL-2 production (53). Massive parallel sequencing determined that some CTCL samples exhibited *PLCG1* mutations, which was linked with increased *NFAT* activation that appeared to drive CTCL proliferation and cell survival (54). scRNA-seq determined that tumor cell gene signatures could accurately predict disease stage and that *FOXP3* overexpression was a primary factor that could predict early disease in SS (55). In a separate study of patients with SS, tumor cells commonly exhibited activating mutations in *CCR4* and *CARD1*, and *ZEB1* was deleted in over half of the patients (2). *AIRE*, which encodes for a protein that functions in central immune tolerance, was upregulated in 58% of malignant cells in SS versus 8.7% of non-malignant cells in one study (56).

Differential mutational burdens may exist between skin resident and circulating tumor cells from the same patient, influencing disease progression. Single-cell sequencing of tumor cells from matched blood and skin samples from patients with L-CTCL revealed that distinct transcriptional signatures exist based on the tissue from which the cells were recovered (57). Expression of genes such as *PDCD1* and *NR4A1* were upregulated in cells isolated from the skin, whereas *KLF2*, *TCF7*, and *SELL* were upregulated in cells isolated from the blood. It is unclear if tissue localization drove epigenetic changes or vice versa. Interestingly, skin-localized tumor cells had higher proliferative activity *ex vivo* as compared to blood-localized cells, which suggested that the skin microenvironment promoted more aggressive malignant expansion than the blood.

## Individualizing CTCL treatment

HTS and scRNA-seq characterization of unique gene expression patterns in CTCL to detect vulnerabilities allow for targeted therapies that minimize morbidity. Targetable tumor biology includes aberrant intracellular signaling pathways and altered protein expression. The high level of heterogeneity of the CTCL transcriptional landscape suggests that personalizing treatment to a patient’s unique tumor biology and treatment response monitoring are important components of high-quality care.

Some gene mutation patterns, most commonly *TP53* loss of function or deletion mutations, are frequently associated with CTCL. Signaling pathway mutations that promote increased T cell survival and activation include increased TCR/CD28 signaling via decreased *PTEN* expression or increased expression of *PLCG1*, *CD28*, and/or *RLTPR*, increased *JAK/STAT* signaling via increased *JAK1* and *STAT3/STAT6* signaling (58, 59), and increased NFκB signaling via increased expression of *CARD11* and *IRF4* (53), among other genes. Researchers have documented epigenetic dysregulation with increased gene methylation (8). Ruxolitinib, a JAK1/JAK2 inhibitor, appears to provide treatment benefit to patients with CTCL-containing mutations promoting JAK/STAT signaling (60).

Malignant CTCL cells often overexpress surface proteins. These include the co-stimulatory receptor CD30 (55), the chemokine receptor CCR4 (2), and the IL-2 receptor CD25, among others. Drugs such as brentuximab vedotin, an antibody-drug conjugate, have been successfully utilized to treat CTCL patients with CD30+ tumor cells (61, 62).

Host immune targets offer therapeutic targets. CTCL tumor cells express higher levels of PD1, and anti-PD1 therapies have shown efficacy in treating this. However, in advanced CTCL, tumor cells may lose PD1 expression via *PDCD1* gene deletion, a process associated with worse patient survival (63). There is no evidence to date, however, that PD1 blockade can induce accelerated progression of CTCL, even in patients with *PDCD1* deletion (64). Blockade of CD47, a protein that suppresses phagocytosis, within skin localized CTCL lesions has shown promise in CTCL treatment (65).

Personalizing CTCL treatments based on tumor mutations and protein expression remains limited by the frequent failure of therapies targeted toward mutated genes. For example, patient responses to brentuximab vedotin are often discordant in relation to measured tumor CD30 expression. Additionally, only a minority of SS patients were found to respond to HDAC inhibitors despite many patients exhibiting HDAC-targetable mutations (66–68). CTCL tumor cells appear to diverge into transcriptionally distinct subsets after HDAC inhibitor therapy (56). These findings may be due to intra-tumoral heterogeneity that permits a fraction of the tumor cells to resist targeted therapies, and so treatment response monitoring is critical.

Accurate monitoring of CTCL treatment response is important to guide clinical decision-making, including determining the appropriateness of de-escalating therapy in patients with excellent disease control or considering the risk/benefit balance of allogeneic stem cell transplant. Tumor cell clone frequency and MF recurrence have been detected via HTS of TCR clones (35). Decreasing numbers and frequencies of clones, as determined by HTS, is associated with effective treatment response, including response to topical and systemic chemotherapy and radiotherapy (69–71).

## Clinical adoption of molecular techniques

Widespread implementation of molecular techniques in CTCL treatment awaits resolution of challenging obstacles, including lack of standardization in assay design, differences in interpretation guidelines, and prohibitive cost. The only widely used molecular technique by clinicians in CTCL treatment may be flow cytometry and PCR-based TCR clonality assays which aid in CTCL diagnosis. HTS and NGS offer increased sensitivity and specificity but remain limited in use to a few academic centers.

Flow cytometry and PCR-based TCR clonality assays may, however, suffer from lower specificity despite offering high sensitivity. PCR-based TCR clonality assay costs are variable. For example, one 2018 study reported a range of $75-450 across four academic medical institutions (72). This study also surveyed dermatopathologists and determined that (1) most respondents had some training in clonality assays and were familiar with CTCL diagnosis and workup, (2), clonality assays were commonly used in the evaluation and diagnosis of CTCL, and (3), clonality assays still play an adjunctive role in CTCL diagnosis and management, with clinical, histopathologic, and immunophenotypic features being the primary determinants that clinicians employ. The authors concluded that this might be due to uncertainty regarding the accuracy of clonality assays.

Researchers have demonstrated that HTS and NGS offer superior sensitivity and specificity compared to PCR in TCR clonality (37, 44). This approach can also identify disease across time. One study estimated that, in the authors’ hands, the cost of HTS of CTCL TCR was about three to four times more expensive than PCR-based assays (37). However, implementing these techniques in patient care remains limited by financial and logistical complications to a few academic medical centers.

## Conclusion

The numerous challenges to diagnosis and therapy posed by CTCL compels continuous exploration of innovative approaches and techniques to optimize management. Improving molecular techniques offer promise for earlier diagnosis, more accurate prognosis, and more effective individualized therapy for affected patients. The development of standardized protocols to inform how to implement and interpret such tests is critical to enable clinicians to utilize these tests in the care of CTCL patients. Decreasing costs and/or increasing reimbursement for the use of molecular techniques is also important for these goals. Finally, deployment of these techniques in research is crucial to improving understanding of CTCL biology and pathogenesis. A promising future for CTCL treatment awaits if these goals are met.

## Author contributions

ML, NB, EM, VL, and AJ were responsible for the conception of the manuscript and reviewed the literature. ML was primarily responsible for drafting the manuscript. ML, NB, RR, EM, VL and AJ revised it critically for intellectual content. All authors approved the manuscript.
